# Supplementary material for: Chromatographic Analysis and Enzyme Inhibition Potential of Reynoutria japonica Houtt.: Computational Docking, ADME, Pharmacokinetic, and Toxicokinetic Analyses of the Major Compounds
Source: Pharmaceuticals (Basel). 2025 Mar 14;18(3):408. doi: 10.3390/ph18030408 (PMC11944761; doi:10.3390/ph18030408)
Supplement: Supplementary file 1 [file pharmaceuticals-18-00408-s001.zip › Pharmaceuticals_Reynoutria_SM1_revised.pdf]

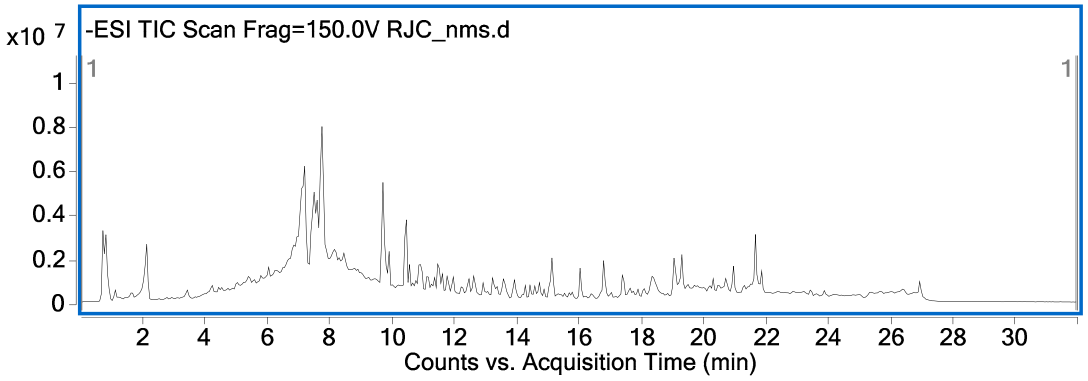

Figure S1. TIC of sample *R. japonica* flower

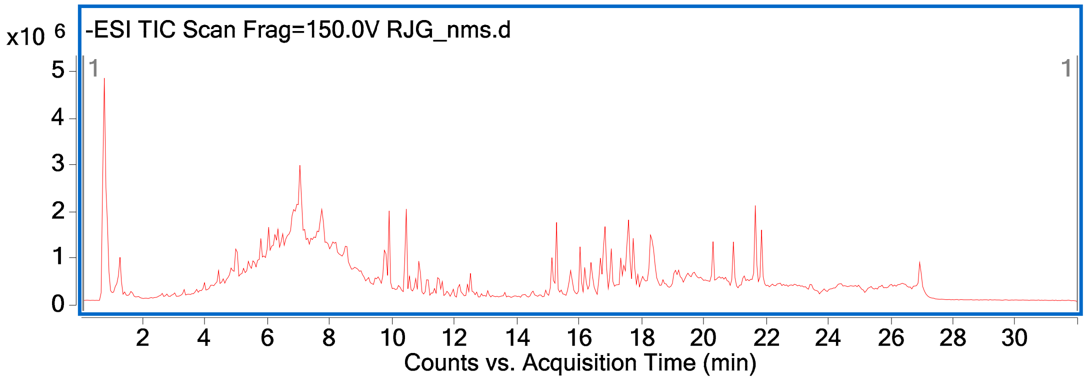

Figure S2. TIC of sample *R. japonica* stem extract

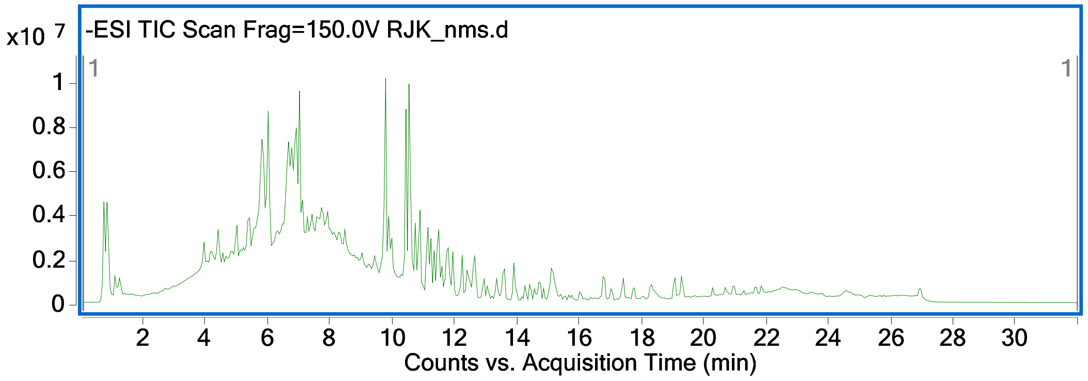

Figure S3. TIC of sample *R. japonica* root extract

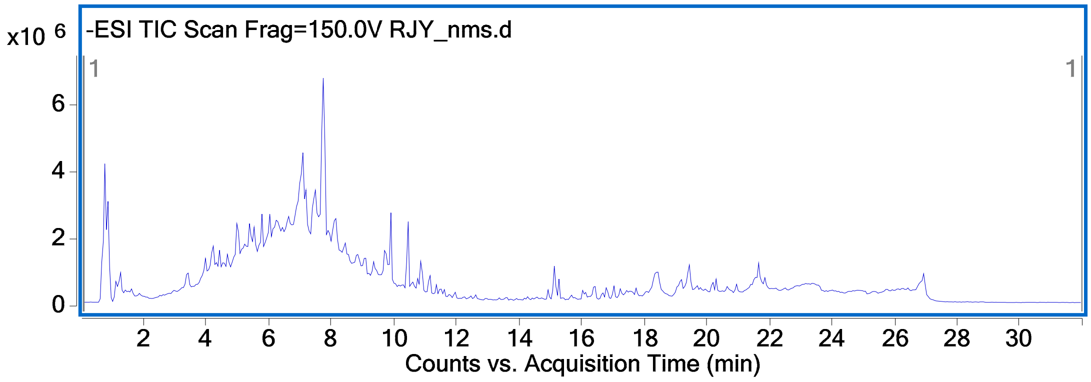

Figure S4. TIC of sample *R. japonica* leaf extract

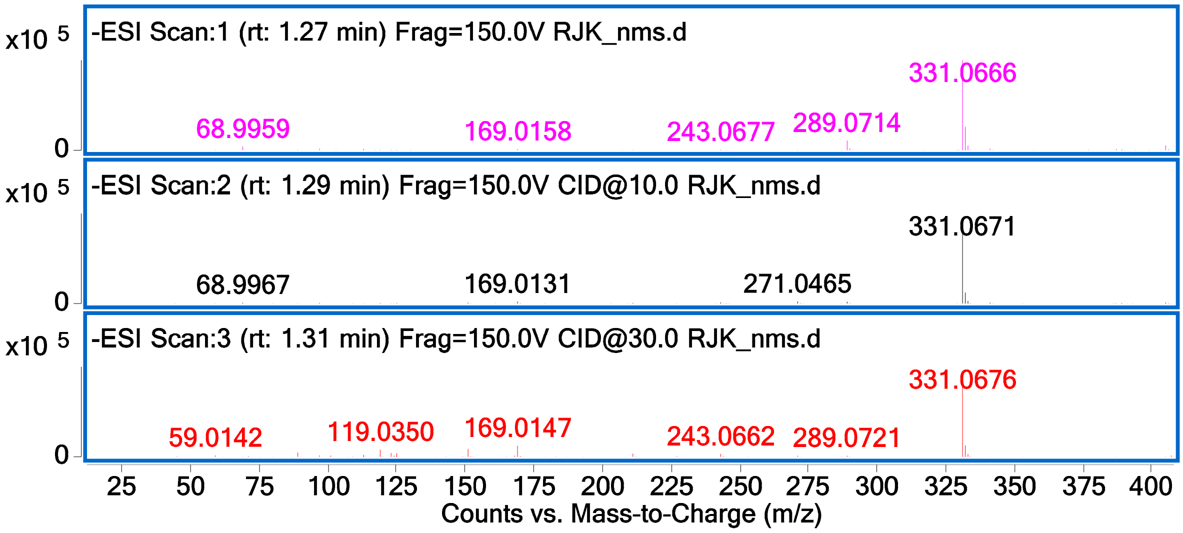

Figure S5. Galloylglucose isomer (331.0666)

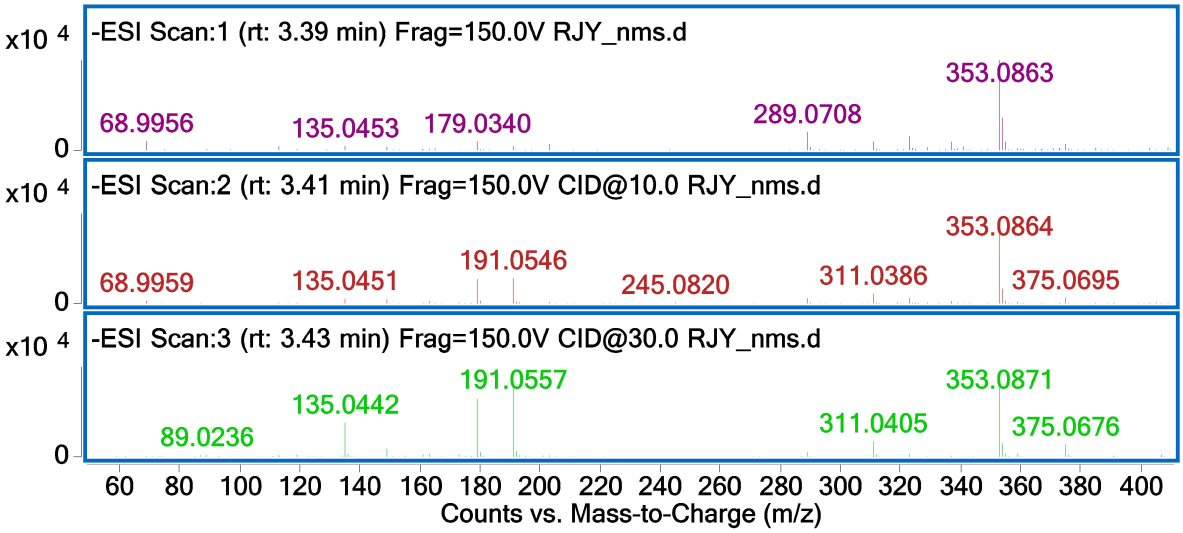

Figure S6. Chlorogenic acid isomer

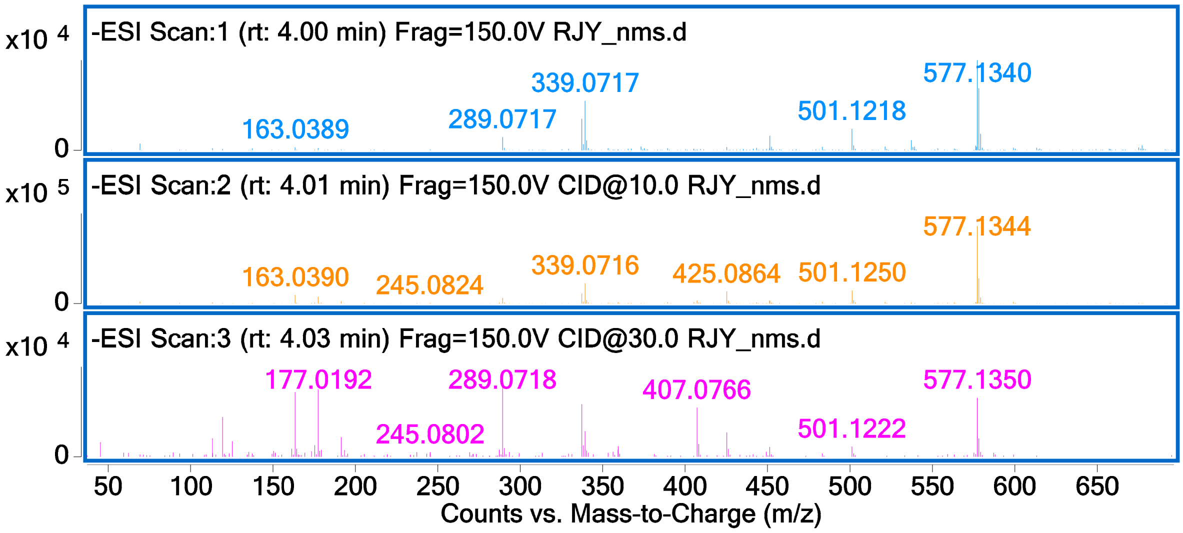

Figure S7. Procyanidin B1 (577.1340)

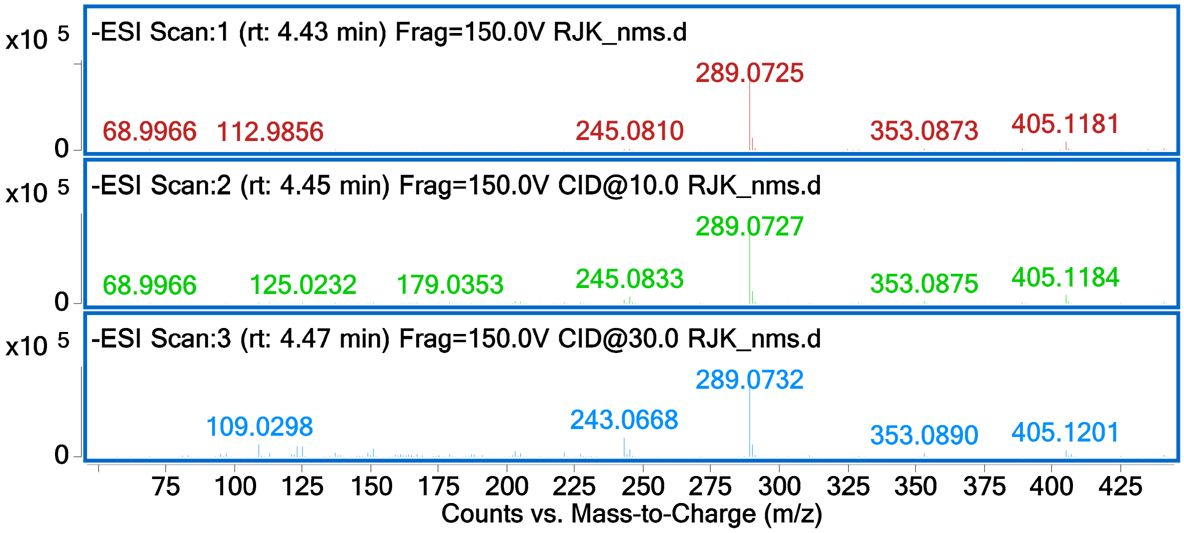

Figure S8. Catechin (289.0725)

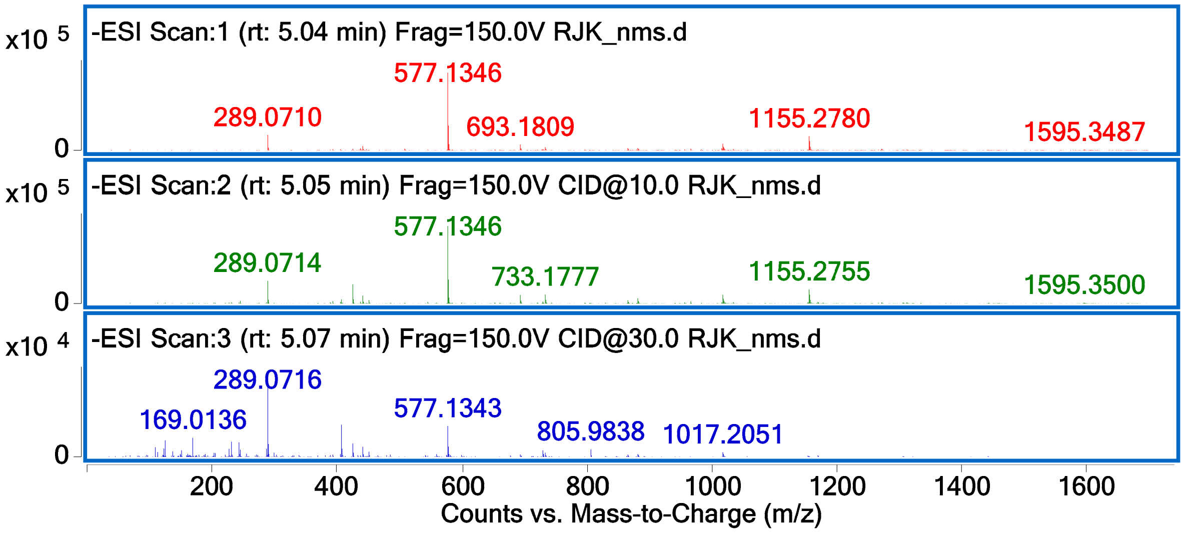

Figure S9. Procyanidin B1 (577.1346)

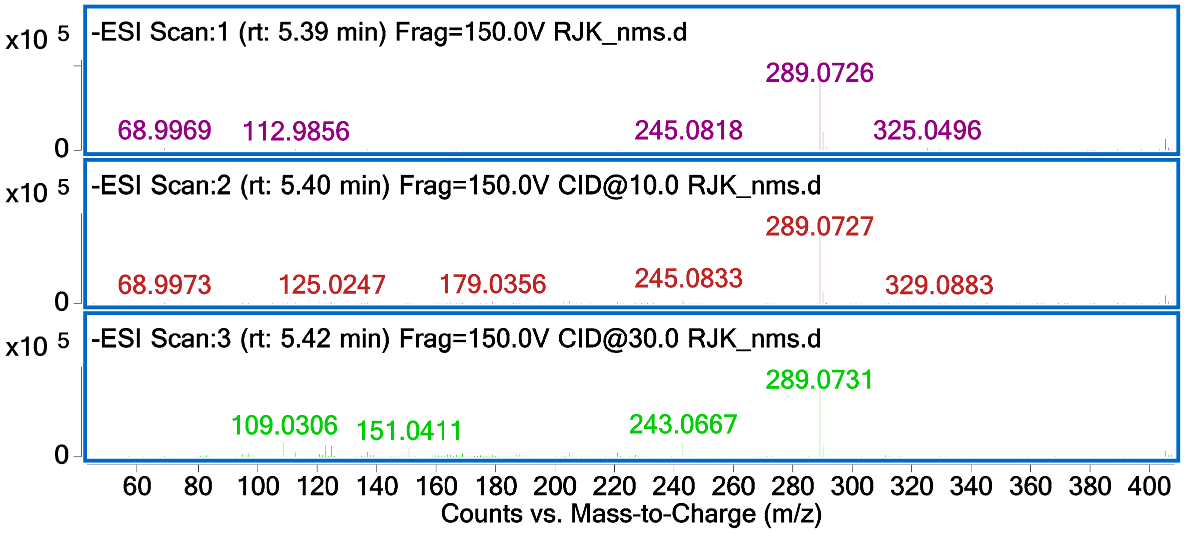

Figure S10. Epicatechin (289.0726)

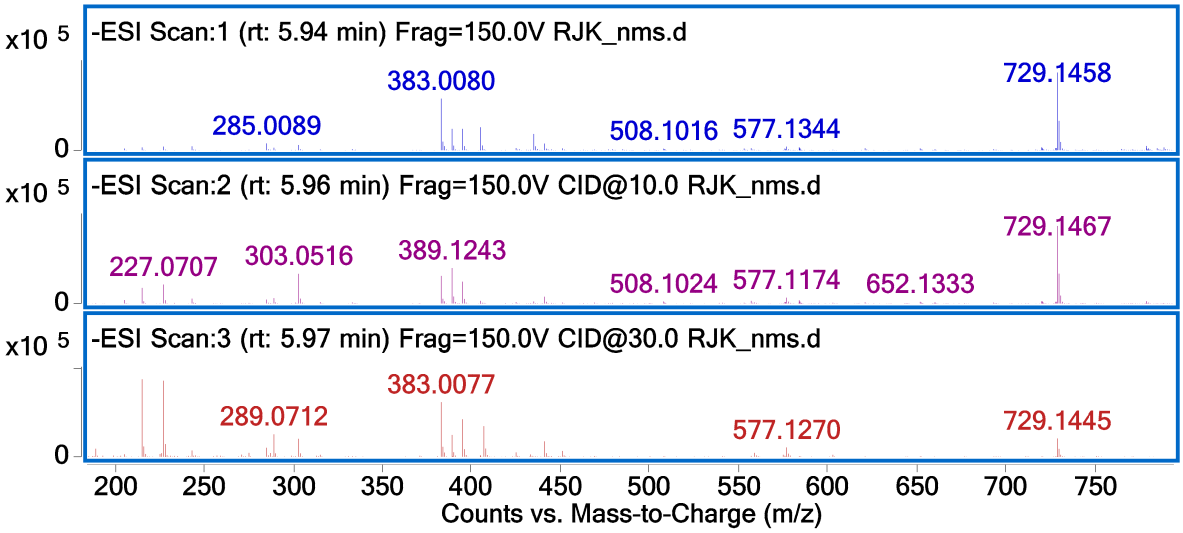

Figure S11. 3-galloylprocyanidin B1/B2 (729.1458)

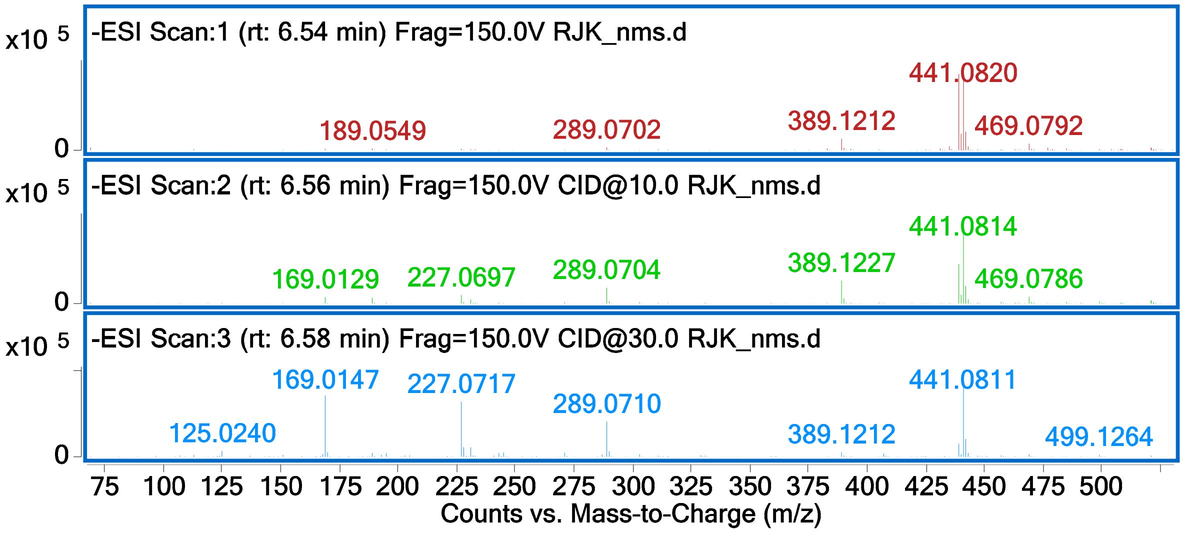

Figure S12. Epicatechin gallate

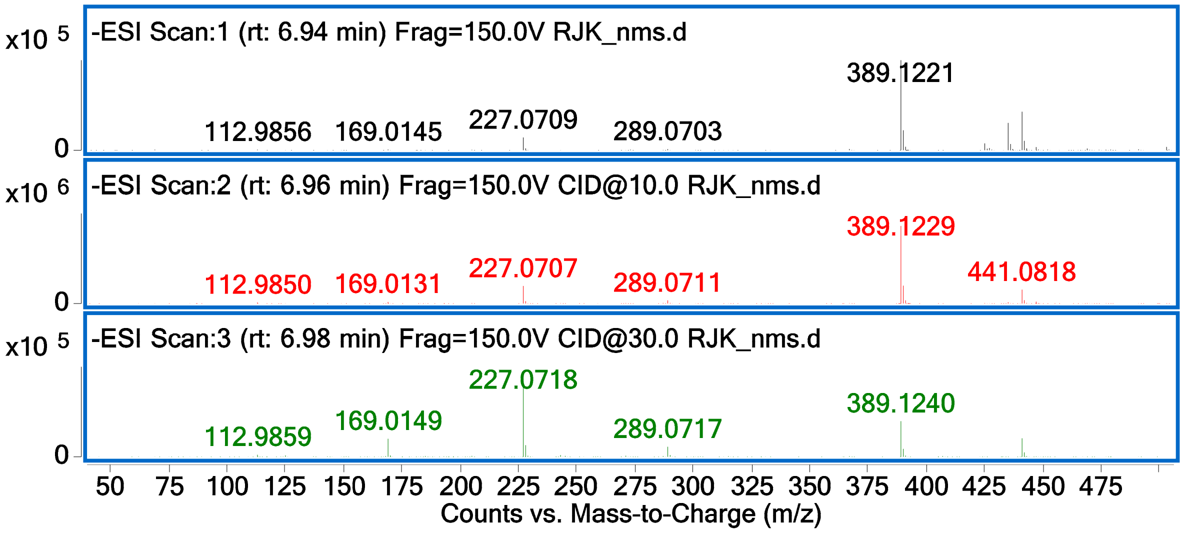

Figure S13. Piceid

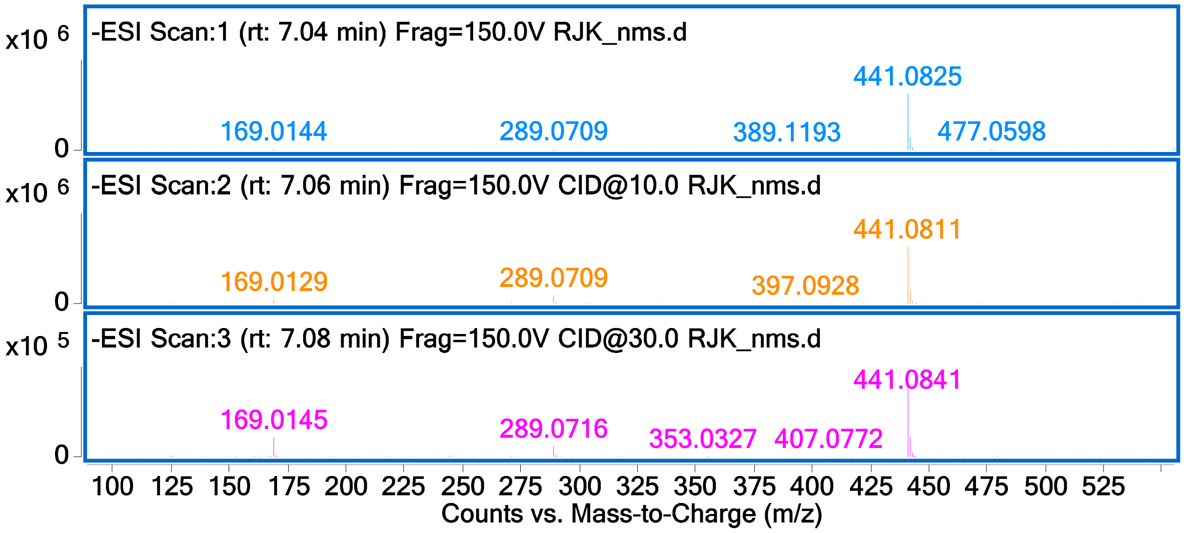

Figure S14. Epicatechin gallate

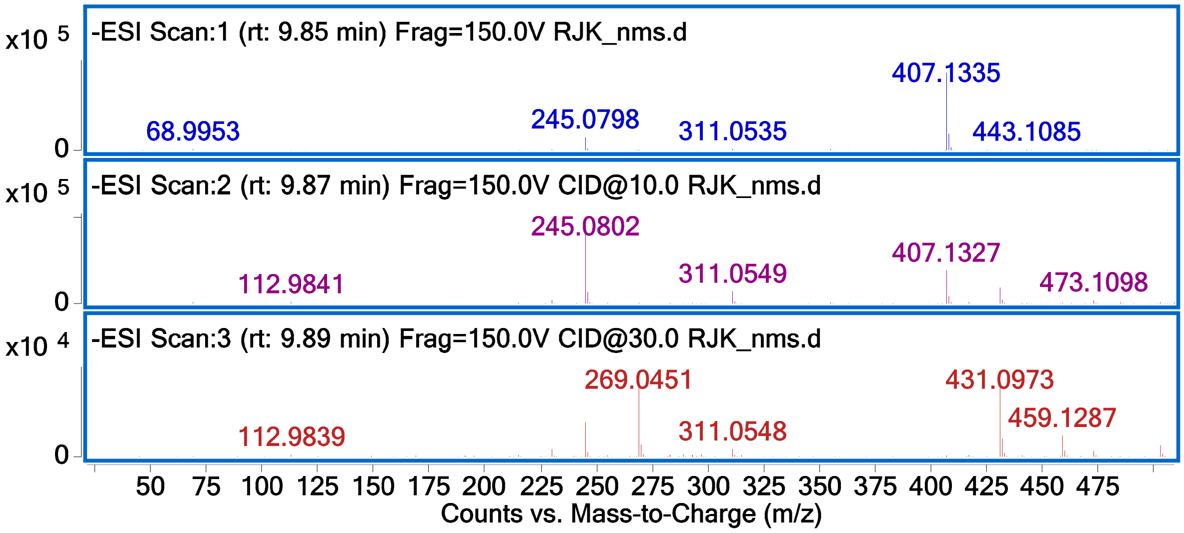

Figure S15. Torachrysone 8-glucoside

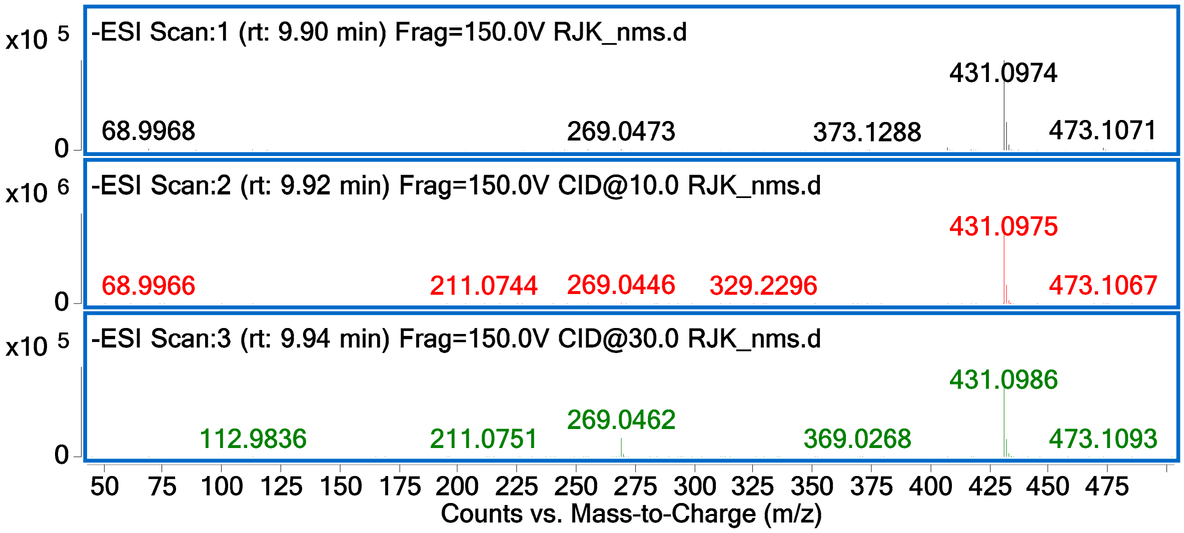

Figure S16. Emodin-6-O-glucoside

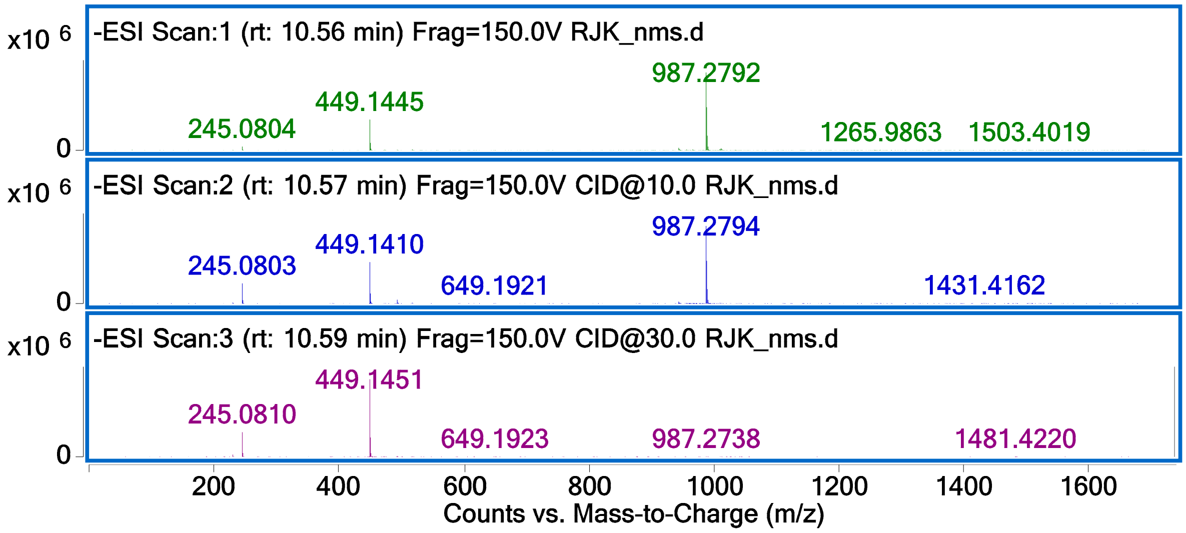

Figure S17. Torachrysone-8-*O*-(acetyl)-glucoside

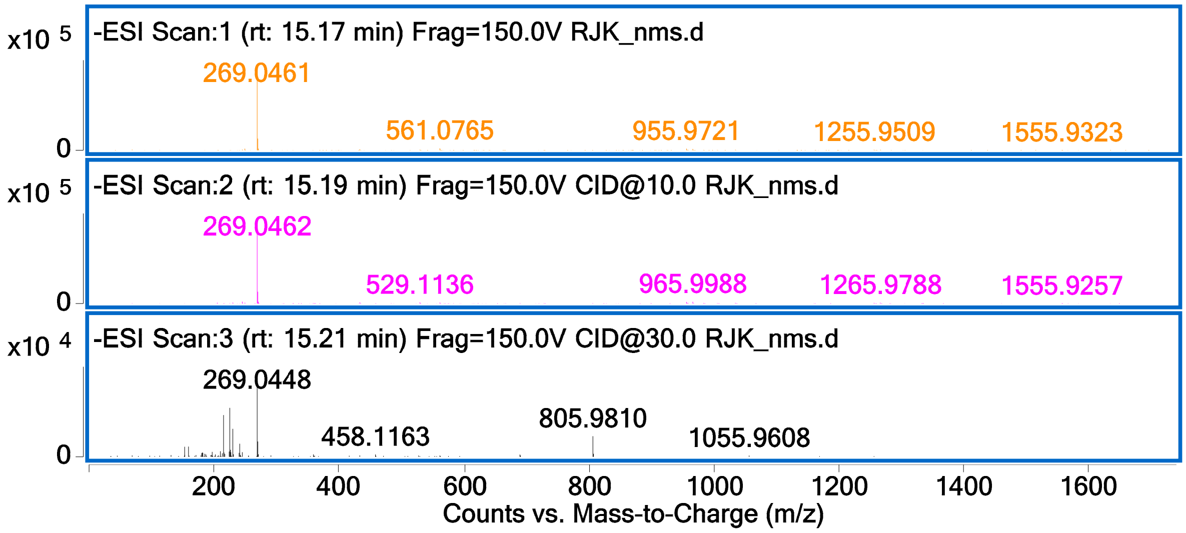

Figure S18. Emodin (269.0461)
